# Translation and Cultural Adaptation into Spanish of the Engagement in Meaningful Activities Survey

**DOI:** 10.1155/2022/4492582

**Published:** 2022-03-28

**Authors:** Fernández-Solano Ana Judit, Merchán-Baeza Jose Antonio, Rodriguez-Bailón Maria, Eakman Aaron

**Affiliations:** ^1^Department of Occupational Therapy, School of Health Sciences, Catholic University of Murcia, Spain; ^2^Research Group on Methodology, Methods, Models and Outcomes of Health and Social Sciences (M3O), Faculty of Health Science and Welfare, University of Vic-Central University of Catalonia (UVIC-UCC), 08500 Vic, Spain; ^3^Department of Physiotherapy (Occupational Therapy), University of Malaga, Málaga, Spain; ^4^Department of Occupational Therapy, Colorado State University Fort Collins, Colorado, USA

## Abstract

**Background:**

Participation in meaningful activities contributes to individual well-being, so it is essential to have specific evaluation tools that can assess this complex construct

**Objectives:**

To create a Spanish version (EDIAS) of the Engagement in Meaningful Activities Survey (EMAS) by following best practices in instrument translation. *Methodology*. The translation was made according to the Principles of Good Practice for the translation and cultural adaptation of patient-reported outcome measures.

**Results:**

The adaptation process involved a meticulous analysis of item equivalence by the expert committee and an expert on the EMAS. Also, harmonization with other translated versions and a cognitive debriefing was carried to assess comprehensibility.

**Conclusion:**

Following best practice, this study has developed the EDIAS, a tool for evaluating participation in meaningful activities adapted to the Spanish context.

## 1. Introduction

People are considered occupational beings with the need to participate in culturally defined occupations. Yet, there must be a balance between personal care, productive and leisure activities, and the presence of meaningful activities to maintain existing skills and to promote health, well-being, and quality of life [1–3].

Meaningful activities fulfil personal or culturally important goals [4, 5]; they are dependent upon our personal values and subjective experiences which are composed of a variety of unique and identifiable aspects associated with our actions, such as pleasure and enjoyment, social connection, and competent completion of tasks [6–8]. Participation in meaningful activities leads to better occupational performance and contributes to individual well-being and the satisfaction of psychological, biological, and cultural needs for a meaningful life, thus improving people's emotional, cognitive, and physical state [4, 5, 8–12].

Given the variety of factors that mediate and influence changes in the meaning of activities, it is essential that evaluation methods are sensitive to changes in these factors over time [6]. Several scales have been developed to evaluate meaningful activities performed during day-to-day life, like the Occupational Value instrument with predefined items (OVal-pd), the Meaningful Activity Participation Assessment (MAPA), or the Engagement in Meaningful Activities Survey (EMAS) [4, 9, 13–16].

The original version of the Engagement in Meaningful Activities Survey (EMAS) uses a 5-point Likert-like self-report scale and open-ended interview questions to construct a profile of a person's activities, including the three activities that most often make the person feel good about himself or herself [4]. The 12 items comprising the EMAS measure engagement in meaningful activities by assessing varied aspects of meaning such as creativity, pleasure, and satisfaction, feelings of competence and control, and a sense of belonging and a capacity for helping others. According to its developers, the EMAS items likely reflect criteria for assessing activity meaningfulness such as “…congruity with one's value system and needs, its ability to provide evidence of competence and mastery and its value in one's social and cultural group” [4]. The EMAS has been shown to have very good convergent and predictive validity relative to measures of meaning and purpose in life and mental health [17–19]. Recently, a 4-category response format of the EMAS has been validated, showing good internal consistency reliability in adult samples [20, 21].

The EMAS has been translated into a multitude of languages such as French, Polish, Malay, Japanese, Persian, Norwegian, Finish, and Lithuanian using, in general terms, a process of forward translation from English to the intended language by different professionals with a good knowledge of English, a focus group to generate a version in the intended language, and finally a backward translation by at least one expert in English [21–23].

In occupational therapy, subjective experience is a central element for understanding the role of doing in the formation of life meaning. It is necessary to discern the varied aspects of meaning in occupation so as to explore how these factors might enrich the quality of our lives [19]. Translating an instrument intended to assess meaning in occupation should therefore be carried out following best practice in the generation of self-report instruments, so that it is sensitive to its unique cultural and clinical contexts. A Spanish version of the EMAS has been developed [24], though the authors had not completed cognitive debriefing with their participants, and therefore, the degree of comprehensibility of the translated questions could not be assessed. In addition, Prat et al. [24] used the 5-category response version of the EMAS which can generate a central tendency bias whereby participants are more likely to choose an intermediate response option [25]. Within the present study, we have adopted for translation of the 4-category response version (i.e., 1 = rarely to 4 = always) as recommended by Eakman [26].

Thus, the aim of this study was to translate the 4-category response version of the EMAS to Spanish and cross-culturally adapt it to suit to the Spanish culture.

## 2. Method

Ethical approval was obtained for the study from the Ethics Committee of the University of Malaga (79-2016-H).

### 2.1. Translation and Cultural Adaptation Process

The adaptation of the EMAS was carried out according to the International Society for Pharmacoeconomics and Outcomes (ISPOR) “Principles of Good Practice.” Those authors recommend guidelines and standards for the translation and cultural adaptation of patient-reported outcome (PRO) measures [27]. This guideline has already been used for the translation and cultural adaptation of other self-reported instruments into the Spanish language [28–30].

**Figure 1:**
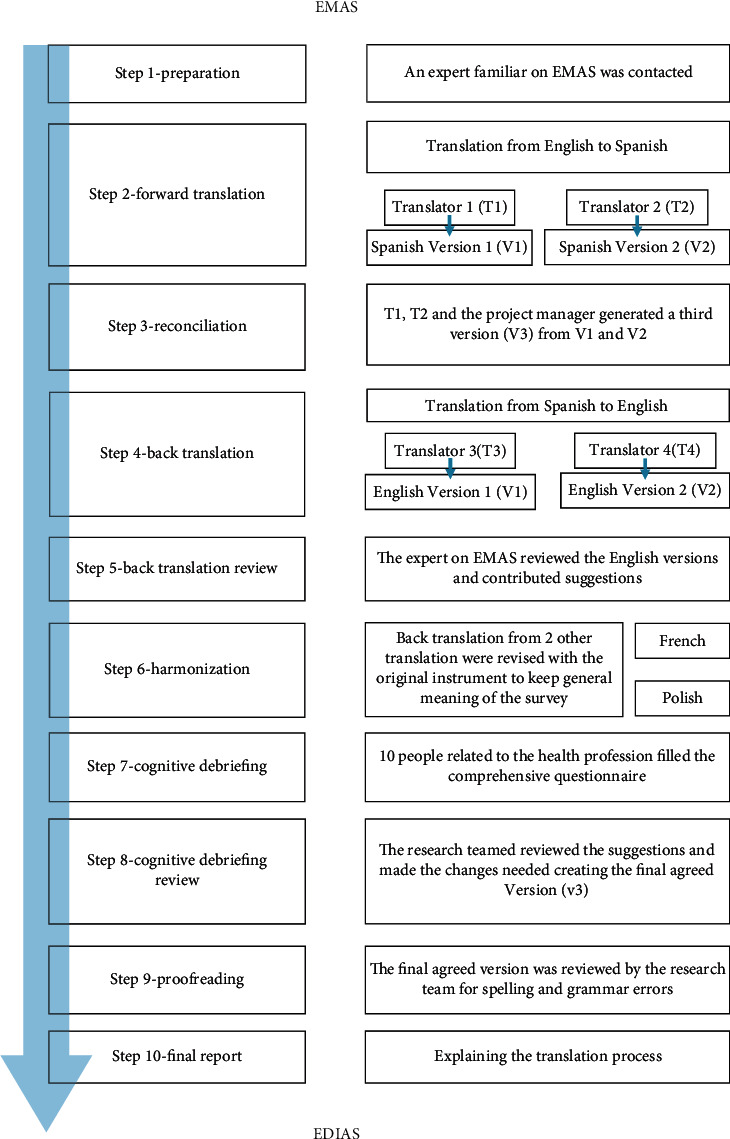


The steps followed for the translation and cultural adaptation of EMAS to Spanish are summarized in the chart shown in Figure 1.

The principles specify ten steps: preparation, forward translation, reconciliation, back translation, back translation review, harmonization, cognitive debriefing, review of cognitive debriefing results and finalisation, proofreading, and final report [27].

- Step 1-preparation. One of the authors of the original assessment tool granted permission to translate the EMAS. The research team contacted an expert familiar with the instrument who agreed to be involved in the translation process. Communication between the research team and EMAS expert was mainly by email
- Step 2-forward translation. Two translators (T1 and T2) produced independent forward translations of the EMAS. One translator was a member of the research team. Both were Spanish occupational therapists, lived in Spain, and had a good knowledge of English. Familiarity with the field of an instrument is important if the instrument uses technical terms that may not be understood by people outside the field [31]. Through conversations, the main concepts of the instrument were explained to the other translator to capture the conceptual meaning of the survey. Two Spanish versions of the instrument were produced (V1 and V2)
- Step 3-reconciliation. The third version (V3) was produced jointly by the two forward translators and the project manager. Over the course of two online meetings, they resolved the discrepancies between the two forward translations and reached a consensus about idioms and preferred phrasing [27]
- Step 4-back translation. Back translations were performed by two translators (T3 and T4) who were not linked to the research team and were blind to the original instrument and its first translation. These translators were native English speakers, had studied English philology, and lived in Spain
- Step 5-back translation review. Both English versions (V1 and V2) of the instrument were sent to the EMAS expert who, after reviewing them, suggested some changes. At this point, we created an expert committee that included a bilingual occupational therapist. The committee reviewed the translations and the comments of the EMAS expert and proposed other translations that, although they were not literal, did capture the full meaning of the original English versions of the problematic items
- Step 6-harmonization. Harmonization of different translations of an instrument is essential to ensure the final translation is culturally appropriate. French [21] and Polish [22] translations of the EMAS were found. The authors of these translations were contacted by email and asked if they would be willing to supply their back translations of the EMAS (Lariviere, June 2, 2019 and Brożek, May 23, 2019). Back translations were revised within the original instrument and ensured that the general meaning of the survey remained intact. No conceptually problematic items were found
- Step 7-cognitive debriefing. We used a cognitive debriefing procedure to assess comprehensibility of the last version and to identify any potential sources of confusion. Respondents rated how well they understood each item from 1 (not understood) to 5 (fully understood) as well as how to rephrase items if they were not fully understood. The questionnaire was sent from an online platform to five professionals working in health-related fields and five students in health-related fields in their final year of studies
- Step 8-cognitive debriefing review. The research team met to review the cognitive debriefing results. Problems identified by the respondents were addressed by adopting the suggested modifications judged as necessary by the expert team
- Step 9-proofreading. The research team checked the final translation for spelling and grammatical errors
- Step 10-final report. A final report explaining the translation process was written and used in the preparation of this paper

## 3. Results

### 3.1. Forward Translation

Table 1 includes examples of how some items were translated into Spanish by both translators (V1 and V2) and the resultant version agreed to by both translators (V3). Table 1 also indicates difficulties found in the translation process, and strategies used to resolve those differences are explained.

### 3.2. Back Translation

English versions of the EMAS from the translators (T3 and T4) were sent to the expert on EMAS who identified three items in the Spanish translation (items 9, 11, and 12) that were problematic because they failed to capture the full meaning of the originals. At this point, we created an expert committee that included a bilingual occupational therapist. The committee reviewed the translations and the comments of the EMAS expert and proposed other translations that, although they were not literal, did capture the full meaning of the original English versions of the problematic items. Item 9 refers to a “feeling of control,” which was initially translated as “*sensación de control*” but was subsequently altered to “*las cosas ocurren tal y como quiero que ocurran*.” This translation focuses on the idea of activities happening the way one wants, rather than on a feeling of being in control of the environment.

Item 11 describes the sensation people usually have when they complete a task. This was not captured by the first translation, “*sentido de satisfacción*,” so it was translated as “*Las actividades que hago me ayudan a alcanzar la satisfacción personal*.”

Item 12, “The activities I do have just the right amount of challenge,” was translated as the “*el nivel de desafío adecuado*” instead of “*el nivel justo de desafío*” because the former is a more common phrase in Spanish.

### 3.3. Cognitive Debriefing

A total of 10 native Spanish speakers were recruited for cognitive debriefing and asked to review the Spanish translation of the EMAS. No major revisions were required because there was full or nearly full understanding for each of the 12 EMAS items. One respondent suggested a revision for item 6 due to limited understanding. The expert team reviewed this recommendation and decided to maintain the original translation because it best reflected the meaning of item 6. The 4-category response options (rarely, sometimes, usually, and always) were fully understood during debriefing, and respondents could easily determine their use in evaluating the level of meaning for each of the 12 EMAS items.

### 3.4. Final Version

The final agreed version (Table 2) was reviewed by the research team for spelling and grammar errors.

## 4. Discussion

The objective of this study was to develop a Spanish version of the 4-category response version of the Engagement in Meaningful Activities Survey (EMAS), which is designed to provide a quick and easy method for evaluating the frequency of varied aspects of meaning in day-to-day activities [26]. Participation in meaningful activities has shown to be crucial for quality of life and mental health both in healthy people [17] and those enduring progressive illness and loss of performance capacities [23, 32], so it is important to have culturally adapted tools such as the EMAS.

The ISPOR method was used to guide the translation and cultural adaptation process. This involved following a sequence of clearly defined steps in order to avoid a number of potential errors [33, 34]. Producing a culturally appropriate adaptation requires more than direct translation [34]. Some items will need to be discussed with the author of the original assessment or with senior researchers who validated the original assessment, in order to decide how best to adapt them so that the meaning of the original is retained [31]. We worked with an EMAS expert who was able to comment on our translations and make suggestions, which provided high methodological quality, thereby creating equivalence between the two versions. For example, in this Spanish version, items 4, 6, and 9 do not start with a translation of “the activities I do…” unlike the Polish and French versions [21, 22]. This approach was taken to best ensure idiomatic equivalence, which maintains the colloquial meaning of the original EMAS items within the local population. In this sense, item 9 (a feeling of control) was a complex construct that required a thorough review between the EMAS expert and the expert committee. As with the French cultural adaptation of the EMAS (Lacroix et al. [21], a “feeling of control” could be interpreted as having control over others. Our approach adopted colloquial Spanish language which preserved the essence of control as having control over oneself and one's life circumstances. Through this process, both a semantic and idiomatic equivalence could be achieved to maintain a colloquial meaning of “control” within our translation [35].

Although in parallel to this work, a translation of the EMAS has been developed in the Spanish context with people with mental illness [24]. The present study stands out for a series of differences. It is worth noting our translation process followed a rigorous method with a series of established steps following the ISPOR method. The translation obtained in Prat et al.'s study is similar to ours, although certain concepts are different, derived from the back translation review with the expert on EMAS we carried out, where the participants indicated expressions that were not entirely understandable such a items 9 “feeling control,” 11 “sense of satisfaction,” and 12 “right amount of challenge” which in Prat et al. are kept in their literal form. As such, we believe the EDIAS may offer a more sensitive and culturally relevant translation of the EMAS. Also, our study used a process of harmonization which involved comparing our back translation with French and Polish translations and with the original version of the EMAS. Harmonization is considered a key objective of the translation and cultural adaption process, ensuring intertranslation validity and allowing for reliable pooling of data from randomized controlled trials [27]. Similarly, in the present study, a bilingual occupational therapist was included in the expert committee, who enhanced the translation process given their knowledge and understanding of key disciplinary concepts in both languages.

The cross-cultural adaptation process used in this study was unique compared to other translations of the EMAS. In many cases, the statistical validation process is emphasized over that of cross-cultural adaptation. As well, only a limited number of necessary steps are carried out, such as back and forward translation as reported by Loh et al. [23] in the Malay version of EMAS or in the Polish version [22]. Although the fundamental value of the validation and analysis of psychometric properties is undeniable, it is also necessary to give weight to translation and cultural adaptation processes in developing a valid assessment tool. A rigorous process of cross-cultural adaptation facilitates achieving adequate psychometric properties of the translated outcome measures [36].

Currently, the EDIAS is in the process of validation with a sample of healthy people, whose data will allow us to test the instrument's psychometric characteristics, such as reliability and construct validity. Among the limitations of this study, it should be mentioned that the cognitive debriefing was carried out with a population without cognitive or comprehension problems. Therefore, future studies should be carried out to verify the degree of understanding of the 12 items of the EDIAS in specific groups of patients with known clinical diagnoses.

### 4.1. Implications

The cross-culturally adapted version of the EDIAS into Spanish can be used, after validation, to determine the frequency of people's engagement in meaningful activities. Likewise, with this study, we offer a complete example of cross-cultural adaptation of an occupational therapy tool following ISPOR guidelines, emphasizing the importance of rigorously complying with the proposed steps.

## 5. Conclusions

This study reports the development of the EDIAS that carefully followed ISPOR guidelines for the translation and cultural adaptation of the English language version of the EMAS into Spanish. Studies are needed to further validate the EDIAS as a measure of engagement in meaningful activities.

## Figures and Tables

**Table 1:** Examples of the forward translation of EMAS from English to Spanish.

Original version	Spanish version (V1)	Spanish version (V2)	Agreed version (V3)
Item 6. The activities I do are valued by other people.	Las actividades que hago son valoradas por otras personas.	Otras personas valoran las actividades que hago.	Otras personas valoran las actividades que hago. *Observations*: we choose the active voice to use the “other people” as the subject of this phrase because we believed it was relevant in the original version.
Item 8. The activities I do give me pleasure.	Disfruto con las actividades que hago.	Las actividades que hago me proporcionan placer.	Las actividades que hago son placenteras. *Observations*: we have chosen this option because it best takes into account the cultural context.
Item 9. The activities I do give me a feeling of control.	Las actividades que hago me dan un sentimiento de control.	Las actividades que hago me dan sensación de control.	Las actividades que hago me dan sensación de control *Observations*: our option here is supported by the use of the word “sensación” which has a more natural or commonly understood meaning.

**Table 2:** Final version of EDIAS.

ESCALA DE IMPLICACIÓN EN ACTIVIDADES SIGNIFICATIVAS			
Aquí debajo hay una lista de afirmaciones sobre las actividades de tu día a día. Por favor, lee cada una de ellas cuidadosamente y elige la respuesta que mejor describe en qué medida cada afirmación es verdadera para ti. Tomate tu tiempo e intenta ser lo más exacto posible			
Escala de respuestas			
1	2	3	4
Casi nunca	A veces	Normalmente	Siempre
			PUNTUACION
1. Las actividades que hago me ayudan a cuidar de mí mismo.			
2. Las actividades que hago reflejan el tipo de persona que soy.			
3. Las actividades que hago expresan mi creatividad.			
4. Me siento satisfecho con los resultados obtenidos en las actividades que hago.			
5. Las actividades que hago contribuyen a que me sienta competente.			
6. Otras personas valoran las actividades que hago.			
7. Las actividades que hago ayudan a otras personas.			
8. Las actividades que hago son placenteras.			
9. Las cosas ocurren tal y como espero que ocurran.			
10. Las actividades que hago me ayudan a expresar mis valores personales.			
11. Las actividades que hago me ayudan a alcanzar la satisfacción personal.			
12. Las actividades que hago tienen el nivel de desafío adecuado.			
Total			

La puntuación se da por la suma de respuestas (desde 1= casi nunca hasta 4= siempre) de los 12 ítems del EMAS que va desde 12-48. La significatividad que perciben las personas en sus actividades puede ser baja (EMAS <29), moderada (EMAS 29-41) o alta (EMAS>41).

## Data Availability

The data are available from the corresponding author upon request.
